# Characterization of Potent ABCG2 Inhibitor Derived from Chromone: From the Mechanism of Inhibition to Human Extracellular Vesicles for Drug Delivery

**DOI:** 10.3390/pharmaceutics15041259

**Published:** 2023-04-17

**Authors:** Glaucio Valdameri, Diogo Henrique Kita, Julia de Paula Dutra, Diego Lima Gomes, Arun Kumar Tonduru, Thales Kronenberger, Bruno Gavinho, Izadora Volpato Rossi, Mariana Mazetto de Carvalho, Basile Pérès, Ingrid Fatima Zattoni, Fabiane Gomes de Moraes Rego, Geraldo Picheth, Rilton Alves de Freitas, Antti Poso, Suresh V. Ambudkar, Marcel I. Ramirez, Ahcène Boumendjel, Vivian Rotuno Moure

**Affiliations:** 1 Graduate Program in Pharmaceutical Sciences, Laboratory of Cancer Drug Resistance, Federal University of Parana, Curitiba 80210-170, PR, Brazil; 2 Laboratory of Cell Biology, Center for Cancer Research, National Cancer Institute, National Institutes of Health, Bethesda, MD 20892-4256, USA; 3 School of Pharmacy, Faculty of Health Sciences, University of Eastern Finland, 70211 Kuopio, Finland; 4 Institute of Pharmacy, Pharmaceutical/Medicinal Chemistry and Tübingen Center for Academic Drug Discovery & Development (TüCAD2), Eberhard Karls University Tübingen, Auf der Morgenstelle 8, 72076 Tübingen, Germany; 5 Microbiology, Parasitology and Pathology Program, Federal University of Parana, Curitiba 81530-000, PR, Brazil; 6 Cell and Molecular Biology Program, Federal University of Parana, Curitiba 81530-000, PR, Brazil; 7 Biopol, Graduate Program in Pharmaceutical Sciences, Federal University of Parana, Curitiba 80210-170, PR, Brazil; 8 Département de Pharmacochimie Moléculaire UMR 5063, Université Grenoble Alpes, 38041 Grenoble, France; 9 Graduate Program in Pharmaceutical Sciences, Federal University of Parana, Curitiba 80210-170, PR, Brazil; 10 Laboratory of Cell Biology, Carlos Chagas Institute, Fiocruz, Curitiba 81310-020, PR, Brazil; 11 Université Grenoble Alpes, INSERM, LRB, 38000 Grenoble, France

**Keywords:** ABCG2 transporter, chromones, inhibitors, extracellular vesicles, drug delivery

## Abstract

Inhibition of ABC transporters is a promising approach to overcome multidrug resistance in cancer. Herein, we report the characterization of a potent ABCG2 inhibitor, namely, chromone **4a** (**C4a**). Molecular docking and in vitro assays using ABCG2 and P-glycoprotein (P-gp) expressing membrane vesicles of insect cells revealed that **C4a** interacts with both transporters, while showing selectivity toward ABCG2 using cell-based transport assays. **C4a** inhibited the ABCG2-mediated efflux of different substrates and molecular dynamic simulations demonstrated that **C4a** binds in the Ko143-binding pocket. Liposomes and extracellular vesicles (EVs) of *Giardia intestinalis* and human blood were used to successfully bypass the poor water solubility and delivery of **C4a** as assessed by inhibition of the ABCG2 function. Human blood EVs also promoted delivery of the well-known P-gp inhibitor, elacridar. Here, for the first time, we demonstrated the potential use of plasma circulating EVs for drug delivery of hydrophobic drugs targeting membrane proteins.

## 1. Introduction

Several strategies can be used for cancer treatment, including chemotherapy, which consists of the administration of cytotoxic drugs. However, cancer cells can show intrinsic resistance or become resistant to the administrated drugs through several mechanisms, such as failure in the activation of programmed cell death, activation of DNA repair pathways and detoxifying systems, alteration of drug targets, decreased cell uptake of drugs and limited cell accumulation of drugs by efflux mediated by ABC transporters [1,2].

The human genome contains 48 genes that encode ABC proteins, organized into seven subfamilies (ABCA-ABCG) [3]. Most of these proteins are membrane transporters that in human are mostly exporters. The functional structure of ABC transporters is based on four domains: two transmembrane domains (TMDs) that recognize the substrates and two nucleotide-binding domains (NBDs), responsible for ATP binding and hydrolysis [4]. The overexpression of ABC transporters on cancer cells probably is the most relevant multidrug resistance (MDR) mechanism, since they can efflux a myriad of compounds with unrelated chemical structures, leading to a decrease in intracellular drug accumulation and impairing the cell response to drug-induced cell death [5,6]. In addition, many ABC transporters share a physiological role by exporting xenobiotics, proteins, metabolic products and lipids in tissues such as the liver, gut, kidney, placenta and blood–brain barrier [6,7].

Among the ABC transporters, two have gained more attention due to their clinical significance in cancer MDR: P-glycoprotein (P-gp), or ABCB1, and ABCG2 [6,8]. P-gp is also known as MDR1 (multidrug resistance protein 1) and it is encoded by the *ABCB1* gene [6]. The ABCG2 protein is encoded by the *ABCG2* gene and the protein is also known by three names: BCRP (breast cancer resistance protein) [9], MXR (mitoxantrone resistance protein) [10] and ABCP (placenta-specific ATP-binding cassette gene) [11]. Among the proposed strategies to overcome MDR mediated by ABC transporters, the use of inhibitors is the most promising [8].

Since P-gp was discovered 46 years ago, several inhibitors have been identified, some of which were validated in animal models and evaluated in clinical trials, such as zosuquidar. However, most of these clinical studies concluded that the use of specific P-gp inhibitors do not improve the chemotherapeutic treatment [12]. Some evidence suggests that the failure of the use of specific P-gp inhibitors was caused by the efflux of drugs mediated by the ABCG2 transporter. Despite the lack of clinical trials using ABCG2 inhibitors, more than a hundred ABCG2 inhibitors have been identified [8], including acridone [13], stilbene [14], chalcone [15], tetrahydroquinoline/4,5-dihydroisoxazole [16], indeno [1,2-*b*]indole [17], porphyrin [18] and chromone [19] derivatives.

**Figure 1:**
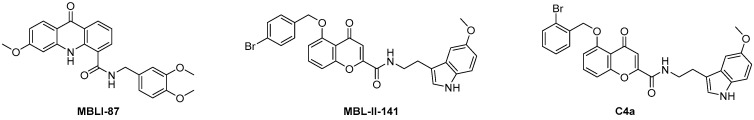
Chemical structure of acridone MBLI-87, chromones MBL-II-141 and chromone **4a** (**C4a**).

Some ABCG2 inhibitors are promising to be used in clinical trials. Among the acridones, the compound named MBLI-87 showed an IC_50_ of 0.45 µM and low cytotoxicity [13]. Subsequently, it was demonstrated that this inhibitor successfully sensitized xenografic tumors overexpressing ABCG2 to irinotecan [20]. Replacement of the acridone moiety by a chromone and the addition of different substituents produced the MBL-II-141 (Figure 1). MBL-II-141 showed a lower IC_50_ (ranging from 0.11 µM [19] to 0.26 µM [21] of ABCG2 inhibition than the acridone derivative MBLI-87 (0.45 µM). Similarly to MBLI-87, the use of MBL-II-141 was also validated in vivo, inhibiting the growth of implanted ABCG2-positive tumors in mice when used in combination with irinotecan [22]. To increase the inhibition potency exhibited by MBL-II-141, further structural optimization led to the rational design of another chromone derivative called chromone **4a** (**C4a**). Notably, moving the halogen on the benzyl group (Figure 1) increased the potency of ABCG2 inhibition (decreased three-fold the IC_50_ value: from 0.26 to 0.086 µM) [21]. However, the precise molecular mechanism of ABCG2 inhibition by **C4a** has not been investigated yet.

In addition, the low water solubility of these compounds limits their administration and transport across physiological barriers, including the solid tumor barrier [23]. Overcoming this challenge is urgent in future studies. Considering the powerful use of lipid nanoparticles (LNPs) as a drug delivery system on the market and in development [24,25,26,27], in this work, we pursued the use of liposomes, an early version of LNPs, to later evaluate the application of human extracellular vesicles (EVs) as vehicles to deliver hydrophobic ABCG2 inhibitors.

## 2. Materials and Methods

### 2.1. ATPase Activity

The ATPase assay was performed according to the protocol described by Ambudkar and co-workers [28]. High-Five insect cell total membrane vesicles overexpressing ABCG2 or P-gp transporter (5 µg/tube) were incubated with assay buffer (2× buffer) composed of 50 mM Tris-HCl, pH 6.8, 150 mM N-methyl-D-glucamine (NMDG)-Cl, 5mM sodium azide, 1 mM ouabain, 2 mM DTT and 10 mM MgCl_2_ in the presence or absence of orthovanadate 0.3 mM at final volume of 100 µL. The combination 2× buffer + protein was incubated with increasing concentration range of **C4a** (0.003 to 1 µM) at 37 °C/20 min in the presence of ATP 5 mM. After incubation, 5% SDS (100 µL) was added to stop the reaction, and 400 µL of P_i_ solution containing 1% ammonium molybdate in 2.5 N H_2_SO_4_, 0.014% of potassium-antimony tartrate and 1% ascorbic acid (200 µL) were used for colorimetric reaction. The absorbance was measured after 10 min incubation at room temperature using the spectrophotometer Ultrospec 3100 pro (Amersham Biosciences, Amersham, UK) at 880 nm wavelength.

### 2.2. Thermostabilization Assay

The thermal stability assay was performed according to previous reports [29]. Briefly, High-Five insect cell total membrane vesicles overexpressing the ABCG2 or ABCB1 transporter at concentration of 3 µg/tube (ABCG2) or 5 µg/tube (P-gp) were incubated with assay buffer composed of 50 mM Tris-HCl, pH 6.8, 150 mM N-methyl-D-glucamine (NMDG)-Cl, 5 mM sodium azide, 1 mM ouabain and 2 mM DTT in the presence or absence of 0.3 mM orthovanadate at final volume of 50 µL. To evaluate the effect of **C4a** over thermal stability, each sample was prepared with 12.5 mM MgCl_2_ or 6.25 mM ATP and incubated at a temperature ranging from 37 to 71 °C for 10 min using a thermocycler C1000 Touch (Bio-Rad, Hercules, CA, USA). After incubation, 10 µL of 25 mM ATP or 50 mM MgCl_2_ (5 and 10 mM final concentration, respectively) was added and incubated at 37 °C/20 min to allow ATP hydrolysis. The reaction was stopped with the addition of 50 µL of P_i_ reagent containing 1% ammonium molybdate, 2.5 N H_2_SO_4_ and 0.014% potassium-antimony tartrate. To evaluate the absorbance, the samples were transferred to a 96-well plate (50 µL/well), then 150 µL of 0.33% sodium ascorbate solution was added. The absorbance was measured after 10 min incubation at room temperature using the microplate reader Spectramax iD3 (Molecular Devices, San Jose, CA, USA). The sensitive activity to vanadate (Vi) was calculated as the difference between the activity in the absence of Vi minus the activity in the presence of Vi at each temperature.

### 2.3. Cell Lines

Cell lines (HEK293-*ABCG*, NIH3T3-*ABCB1* and BHK21-*ABCC1*) were kept at 37 °C in the presence of 5% CO_2_ in DMEM high glucose (Gibco, Sao Paulo, Brazil) supplemented with 10% Fetal Bovine Serum (Gibco, Sao Paulo, Brazil) and 1% antibiotic (penicillin/streptomycin–Hyclone Laboratories, Logan, UT, USA). The stably transfected cells were kept with specific selection agent for each ABC transporter: ABCG2–G418 0.75 mg/mL, P-gp–Colchicine 60 ng/mL, MRP1–Methotrexate 0.1 mg/mL. The cells HEK293-*ABCG2*, NIH3T3-*ABCB1* and BHK21-*ABCC1*, overexpressing ABCG2, P-gp and MRP1, respectively, were kindly provided by Dr. Attilio Di Pietro (IBCP, Lyon, France).

### 2.4. Cell-Based Transport Assay by Flow Cytometry

Cells were seeded into 24-well plates at a density of 1 × 10^5^ cells/well and incubated for 48 h at 37 °C and 5% of CO_2_. When the confluence reached 90–95%, the medium was removed and the cells were exposed to inhibitors and substrates for 30 min at 37 °C. Then, the cell monolayer was washed with PBS at 37 °C, trypsinized and resuspended with 300 µL of cold PBS. The intracellular fluorescence was recorded by flow cytometry (FACS Calibur–Becton Dickinson, San Diego, CA, USA). At least 10,000 events were acquired. The complete inhibition that corresponds to 100% of inhibition was achieved using reference inhibitors, such as Ko143, GF120918 and verapamil. The inhibition percent was calculated as follows: % of inhibition = ((*C* − *S*))/((*I* − *S*)) × 100, where “*C*” represents the treatment with substrate + compound in test, “*S*” represents the treatment with substrate alone and “*I*” represents the treatment with substrate + reference inhibitor.

### 2.5. Cell-Based Transport Assay by Confocal Microscopy

Cells were seeded into 24-well plates at a density of 1 × 10^5^ cells/well containing coverslips for microscopy and incubated for 48 h at 37 °C and 5% of CO_2_. Cells that adhered to the coverslips were treated with **C4a** and hoechst 33342 for 30 min at 37 °C. Then, the cell monolayer was washed with PBS at 37 °C and the coverslips removed from the plate and placed on slides for microscopy. The slides were then read in a confocal microscope Nikon A1R MP + (NIKON, Tokyo, Japan) using an oil-immersed 40× objective (with the numerical aperture of 1.15). A laser of 405 nm was used for excitation and the fluorescence emission was recorded using a bandpass filter of 425–475 nm. The software Nis Elements 4.20 (NIKON, Tokyo, Japan) was used for visualization of the images.

### 2.6. D3 Shift Assay

HEK293-*ABCG2* cells were seeded into 24-well plates at a density of 2 × 10^5^ cells/well. When the confluence reached 90–95%, the medium was removed and the cells were exposed to inhibitors for 30 min at 37 °C. Then, the cell monolayer was washed with PBS at 37 °C, trypsinized and resuspended with 300 µL of PBS. Cells were centrifuged (1000× *g* for 3 min). The pellet was resuspended in 100 μL of PBS/BSA (40 μg/mL of BSA) and the primary antibody (mouse anti-human CD338 Clone 5D3, BD Pharmingen, San Diego, CA, USA) was added (1:100) for 30 min at 37 °C. Cells were centrifuged (1000× *g*, 3 min), the supernatant removed and the pellet resuspended with 100 μL of PBS containing the secondary antibody (goat anti-mouse conjugated with Phycoerythrin, BD Pharmingen, San Diego, CA, USA) at 1:200 for 30 min at 37 °C. Then, cells were centrifuged, the supernatant removed and the pellet was resuspended in 300 μL of PBS. The analysis was performed using a FACS Calibur (Becton Dickinson, San Diego, CA, USA).

### 2.7. Molecular Modelling

The preparation of the systems and docking calculations were performed using the Schrödinger Drug Discovery suite for molecular modelling (version 2019.4). The ABCG2 crystal structures (PDB ID: 6VXJ, bound to SN38, resolution 4.0 Å [30] and PDB ID: 6VXF apo structure, resolution 3.50 Å) was obtained from the Protein Data Bank (PDB, www.rcsb.org). The structure was chosen since it is one of the cryo-EM structures with high-resolution reported. ABCG2 structure was prepared with the Protein preparation wizard [31] to fix protonation states of amino acids residues, adding hydrogens and also fixing missing side-chain atoms. Missing residues for the protein (residue numbers 47–60 and 355–369) were predicted using Prime protein preparation module [32]. Homology models were generated for both the crystal structures to predict the remaining missing residues 302-327, using the respective prepared crystal structures as templates. We have used both chain A and B to build the model, Energy based method was used for building the model, with options to retain rotamer conformations for conserved residues of Prime. 

Compounds Ko143 and chromone **4a** were drawn using maestro and prepared using LigPrep [33] to generate the three-dimensional conformation, adjust protonation state to physiological pH (7.4), and calculate the partial atomic charges, with the force field OPLS3e [34]. Docking studies with the prepared ligands were performed using Glide [35,36] (Glide V7.7), with the flexible modality of Induced-fit docking with extra precision (XP), followed by a side-chain minimization step using Prime [37]. Ligands were docked within a grid around the co-crystallized ligand with the centroid near the Phe439(A) and Phe439(B), as to represent Site 1 in between the subunits. For each ligand 10 docking poses were generated. Selected docking poses were further validated by molecular dynamics simulation, where ligand stability within the proposed pocket and its interactions were evaluated.

Molecular dynamics simulations were carried out using Desmond [38] engine with the OPLS3e force-field [34,39]. Protein was embedded within a DMPC lipid pre-generated from Maestro, using the System Builder, using ABCG2’s alpha-helices to orient it. The system was placed in a cubic box with 13X13X15 Å from the box edges to any atom of the protein, using PBC conditions, and, filled with TIP3P [40] water. Systems were equilibrated as previously described [18]. After minimization and relaxing steps, we proceeded with five independent 200 ns runs with randomly generated seeds (in total 1 µs per system). Trajectories and interaction data are available on Zenodo repository (10.5281/zenodo.7147613). Docking models of the ligands, binding sites and amino acids interactions with the proteins (ABCG2 and P-gp) from relevant frames of the simulation were visualized using PyMOL 2.5.2.

### 2.8. Preparation of Liposomes

Liposomes were prepared with distearoyl phosphatidylcholine (DSPC) and dioleoylphosphatidylethanolamine (DOPE). A total of 20 mg of DSPC, 1.86 mg of DOPE and 5.3 mg of **C4a** (that corresponds to 1 mM) were solubilized in 10 mL of dichloromethane in a ground-glass round-bottomed flask. This solution was purged with N_2_ for 2 min for partial removal of O_2_. By rotary evaporation, the volatile solvent was completely removed, forming a film on the wall of the flask. The latter was submerged in an oil bath at 70 °C and 10 mL of PBS was added. The flask was kept under these conditions for 1 h with vigorous magnetic stirring. The product was sonicated for 3 min to disrupt the aggregates, homogenizing the size of the formed liposomes. This preparation was named Lip-**C4a**.

### 2.9. Extracellular Vesicles from Giardia Intestinalis

The trophozoites of *Giardia intestinalis* (also known as *Giardia lamblia*) were cultured in TYI-S-33 medium in 15 mL tubes at 37 °C and 5% of CO_2_ until confluence of 1 × 10^6^ trophozoites/mL. After 15 min on ice, cells were centrifuged at 600× *g* for 5 min at 4 °C. The pellet was resuspended in 6 mL of TYI-S-33 medium, and cells were centrifuged at 600× *g* for 5 min at 4 °C. The pellet was resuspended in 6 mL of TYI-S-33 and cells were incubated with 1 M of CaCl_2_ at 37 °C for 1 h. After CaCl_2_ exposure, cells were centrifuged at 600× *g* for 5 min and the supernatant was collected in microtubes, which were centrifuged twice at 4000× *g* for 30 min (the supernatant was collected, and the pellet discarded). Then, the supernatant was ultracentrifuged at 100,000× *g* for 1.5 h. Protein concentration was quantified using the micro-BCA protein assay (Thermo Scientific, Waltham, MA, USA). For this study, 4 μg of EVs was incubated with **C4a** (or hoechst 33342) for 30 min at room temperature. After, EVs containing **C4a** (or hoechst 33342) were ultracentrifuged at 100,000× *g* for 1.5 h to remove the solvent DMSO. The pellets containing EVs were resuspended with 100 µL of PBS.

### 2.10. MTT Assay

HEK293 WT (wild type) and HEK293-*ABCG2* cells were seeded (2.5 × 10^4^ cell/well) into a 96-well plate and incubated for 24 h. Cells were concomitantly treated with EVs *G. intestinalis*-**C4a** (0.1, 1 and 10 μM) and SN-38 (10 nM) for 72 h. After this period, the medium was removed, cells were washed with PBS (100 μL) and incubated with MTT solution (100 μL of solution 0.5 mg/mL in PBS) for 4 h. Then, the solution was removed, and the formazan crystals were dissolved with 100 μL of ethanol/DMSO (1:1). The absorbance was measured using a microplate reader at 595 nm (Bio-Rad iMark, Hercules, CA, USA).

### 2.11. Extracellular Vesicles from Human Blood

This study was approved by the Ethics Committee of SCS/UFPR (CAAE 51428521.2.0000.0102). Human blood (9 mL) was collected in tubes containing 8 mM of EGTA and centrifuged at 425× *g* for 10 min at room temperature. Human plasma (~4 mL) was separated in a new tube. In the tube containing blood cells (~5 mL), the same volume of RPMI (~5 mL) was added. The material was homogenized and centrifuged at 425× *g* for 10 min at room temperature. The supernatant was discarded, and the pellet of cells was centrifuged again at 425× *g* for 10 min, then, the supernatant was discarded, and the pellet was suspended in 5 mL of RPMI containing 2 mM of CaCl_2_. After 1 h of incubation at 37 °C, the material was centrifuged at 425× *g* for 10 min at 10 °C. Additionally, the tube containing the human plasma was also centrifuged at 425× *g* for 10 min at 10 °C. The supernatant from both tubes (human cells and plasma) was centrifuged at 4000 *g* for 10 min at 10 °C. The supernatant was separated and centrifuged at 11,000 *g* for 120 min at 10 °C. The supernatant was discarded and the pellet containing the vesicles was suspended in 60 µL of PBS. The protein concentration was quantified by Bradford assay. For each condition, 10 µL of the vesicles in PBS was diluted by adding 190 µL of PBS. The diluted vesicles were incubated with different concentrations of **C4a** or elacridar (GF120918) for 30 min at room temperature. Then, the material was centrifuged at 11,000 *g* for 120 min at 10 °C. The supernatant was separated in a new tube and the pellets containing the vesicles were suspended in 50 µL of PBS. HEK293-*ABCG2* or NIH3T3-*ABCB1* cells were treated with either supernatant (200 µL) + 100 µL DMEM or vesicles (50 µL) + 250 µL DMEM. After, the ABCG2 substrate hoechst 33342 (5 µM) or the P-gp substrate rhodamine 123 (10 µM) were added and incubated for 45 min at 37 °C. Then, the procedure was performed as described in the item “cell-based transport assay by flow cytometry”.

## 3. Results

### 3.1. Interaction of Chromone ***4a*** (***C4a***) with ABCG2 and P-Glycoprotein

**Figure 2:**
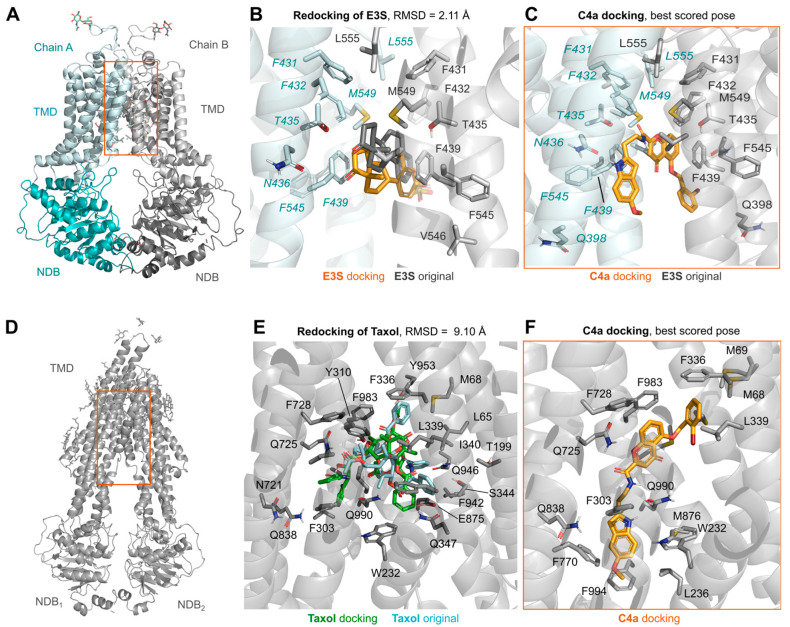
In silico interaction of **C4a** with ABCG2 and P-gp. Molecular docking analysis in human ABCG2 (PDB: 6HCO) (**A**–**C**) and P-gp (PDB: 6QEX) (**D**–**F**). Amino acid residues are colored according to the atom involved in the interaction (carbon, light grey (chain A of ABCG2 and P-gp) and pale blue (chain B of ABCG2); oxygen, red; nitrogen, blue). (**A**,**D**) display an overview of the transporter structure, highlighting the position of the docking pose box as an orange square. The original conformation of substrates estrone-3-sulfate (E3S) for ABCG2 display small conformational changes when compared to the highest scored docking pose from molecular docking (**B**), while redocking of taxol within P-gp is much more expressive (**E**). Best scored pose of the interaction of **C4a** with ABCG2 (**C**) and P-gp (**F**).

Preliminary docking of **C4a** on ABCG2 suggested binding within the transmembrane region (Figure 2A–C), specifically within the substrate-binding pocket (see MD simulations below). A comparison between the binding of ABCG2 substrate estrone-3-sulfate (E3S) and **C4a** revealed an overlap of the binding site (Figure 2A–C). The interaction of both compounds on ABCG2 shared several amino acid residues, such as N436, F439 and L555 (Figure 2A–C). Considering that many P-gp inhibitors bear a chromone moiety, molecular docking analyses were performed on P-gp (Figure 2D–F). Interestingly, despite the poor redocking results, the large P-gp pocket could accommodate **C4a** with high predicted affinity, resulting in a reasonable pose. In addition, the **C4a** binding site on P-gp was slightly different from the binding site of the taxol substrate.

**Figure 3:**
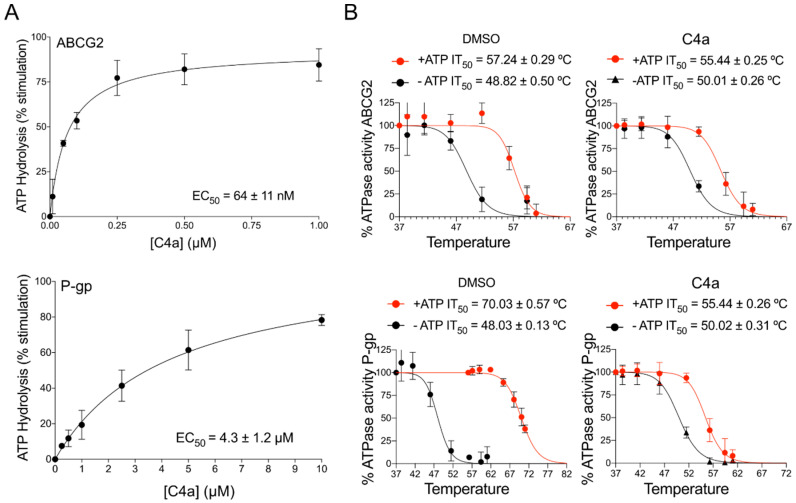
Membrane-based studies of the interaction of **C4a** with ABCG2 and P-gp. (**A**) Effect of **C4a** on ATPase activity using High-Five ABCG2 and P-gp total membrane vesicles. EC_50_ value is the concentration giving a half-maximal effect. (**B**) Thermostabilization assay with **C4a** at saturating concentration of 10 μM on the ATPase activity of ABCG2 and P-gp. The data are the mean ± SD of three independent experiments performed in duplicate.

To confirm the computational analysis, the effect of **C4a** on the ATPase activity of ABCG2 and P-gp was investigated using total membrane vesicles of insect (High-Five) cells overexpressing ABCG2 or P-gp. As shown in Figure 3A, **C4a** stimulated the basal ATPase activity, however, the affinity toward ABCG2 was 67-fold higher (EC_50_ 64 nM) than for P-gp (EC_50_ 4.3 µM). Additionally, the effect of Ko143 on ATPase activity was investigated. In contrast to **C4a**, Ko143 inhibited the ATPase activity, showing an EC_50_ value of 30 nM (Figure S1).

To further investigate the interaction of **C4a** with ABCG2 and P-gp using a membrane-based approach, a thermal stability assay was performed. This experiment was performed in two conditions: Absence versus presence of ATP. As shown in Figure 3B, **C4a** triggered a mild effect on ABCG2, since the IT_50_ (temperature that reduces 50% of the ATPase activity) values in presence or absence of **C4a** were very similar. In contrast, **C4a** stabilized the inward-open conformation of P-gp, resulting in the loss of ATP-induced dimerization of NBDs and consequently preventing thermostabilization (Figure 3B).

### 3.2. Cell-Based Studies of the Interaction of Chromone ***4a*** (***C4a***) with ABCG2

**Figure 4:**
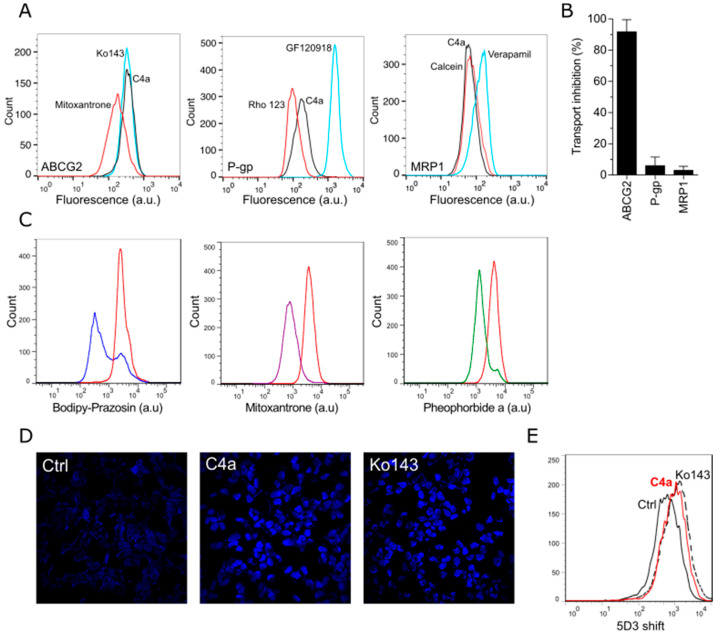
Cell-based studies of the interaction of **C4a** with ABCG2. Transport assay using stable transfected cell lines overexpressing ABCG2, P-gp and MRP1. For ABCG2 transporter, mitoxantrone (10 µM) and Ko143 (1 µM) were used as substrate and reference inhibitor, respectively. For P-gp transporter, rhodamine 123 (10 µM) and elacridar (1 µM) were used as substrate and reference inhibitor, respectively. For MRP1 transporter, calcein-AM (0.2 µM) and verapamil (35 µM) were used as substrate and reference inhibitor, respectively. **C4a** was used at 10 µM. (**A**) Representative histograms obtained by flow cytometry. (**B**) Data of mean ± SD of three independent experiments performed in duplicate. (**C**) Cell-based ABCG2 inhibition transport assay by flow cytometry using different substrates: bodipy-prazosin (0.2 µM), mitoxantrone (10 µM) and pheophorbide *a* (10 µM). (**D**) Cell-based ABCG2 inhibition transport assay by confocal microscopy using hoechst 33342 (2 µM) as substrate. (**E**) 5D3 shift assay by flow cytometry using **C4a** (10 µM) and Ko143 (2 µM).

To investigate the selectivity of **C4a** on transport inhibition of ABCG2, a cell-based transport assay using stably transfected cells overexpressing ABCG2 (HEK293-*ABCG2*), P-gp (NIH3T3-*ABCB1*) and MRP1 (BHK21-*ABCC1*) was used. As shown in the histograms obtained by flow cytometry (Figure 4A), **C4a** at 10 µM completely inhibited the mitoxantrone efflux mediated by ABCG2 (left histogram). However, no significant inhibition effect of **C4a** was observed in P-gp and MRP1 transporters, since **C4a** at 10 µM did not inhibit the rhodamine 123 efflux mediated by P-gp (middle histogram) and the calcein efflux mediated by MRP1 (right histogram). As shown in Figure 4B (triplicate data), **C4a** is a selective inhibitor of the ABCG2 transporter.

To confirm the ABCG2 functional inhibition, the same cell-based approach was used using different substrates, including bodipy-prazosin, mitoxantrone, pheophorbide-*a* and hoechst 33342. The transport inhibition of bodipy-prazosin, mitoxantrone and pheophorbide-*a* was investigated by flow cytometry, while the transport inhibition of hoechst 33342 was studied by confocal microscopy. As shown in Figure 4C,D, **C4a** at 10 µM inhibited the efflux of all these chemically unrelated fluorescent ABCG2 substrates. Additionally, to endorse the inhibition feature of **C4a** on ABCG2, the 5D3 shift assay was performed. This assay consists of the use of the anti-ABCG2 conformational antibody clone 5D3 that recognizes an extracellular loop of this protein. The results demonstrated that **C4a** increased the binding of 5D3, such as the reference inhibitor Ko143 (Figure 4E).

### 3.3. Molecular Dynamic Simulations of ABCG2 in Presence of Chromone (***C4a***)

**Figure 5:**
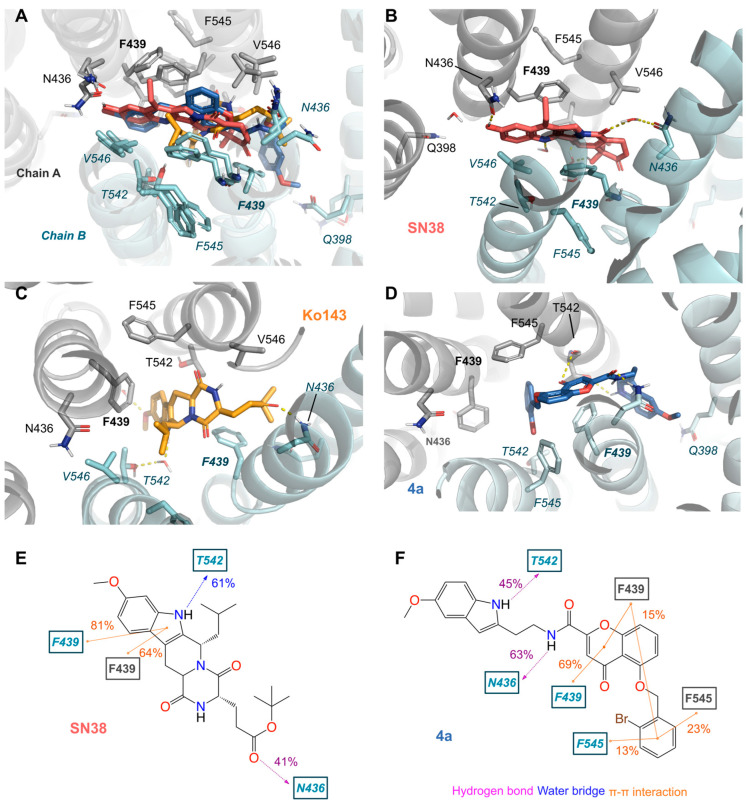
Molecular dynamic simulations: (**A**) Representative snapshots of the last frames for molecular dynamics simulations highlight the superposition of all poses. (**B**) SN38 substrate—red, (**C**) Ko143 inhibitor—orange and (**D**) **C4a**-blue. ABCG2 amino acid residues are colored according to the atom involved in the interaction (carbon, light grey (chain A) and pale blue (chain B); oxygen, red; nitrogen, blue). Hydrogen bond interactions and water bridges are represented by dashed yellow lines. (**E**,**F**) Two-dimensional representation of the interaction frequency for SN38 (**E**) and **C4a** (**F**) observed along with the molecular dynamics simulations. Each number represents the mean of at least five independent simulations (5 × 200 ns).

To further explore the potential binding modes of **C4a** on ABCG2, molecular dynamic (MD) simulations were performed using ABCG2 cryo-EM structures. **C4a** simulations were compared against apo and relevant ligands (Ko143 and the substrate SN38). All simulated compounds share common hydrophobic contacts and π-π interactions, mainly with the V546 and F439, respectively (Figure 5 and Figure S2A–C), with similar interaction frequency for SN38 and **C4a** (Figure 5E,F). The original conformation of the substrate SN38 had little change along with the simulations (Figure S2D); however, both inhibitors Ko143 and **C4a** explored a larger conformational space, as observed by their RMSD ligands values (Figure S2E,F).

The binding of SN38, Ko143 and **C4a** induces small conformational changes in the transmembrane binding domains (TMD) and the nucleotide binding domains (NBD). Simulations with these ligands stabilized the ABCG2 structure in the TMD-closed conformation (closed inward-facing conformation), by blocking the gap between subunits as observed by the smaller mean distance between F439 in comparison to the apo structure (Figure S3A–C). Additionally, the NBD-open conformation is also stabilized in the ligand-bound conformations; in comparison with the apo structure counterpart (Figure S3A–C), little conformational changes were observed in the NBD (Figure S4), which could be attributed to artefacts from the highly flexible residues in these regions.

### 3.4. Lipid-Nanoparticles-Based Delivery of Chromone (***C4a***)

**Figure 6:**
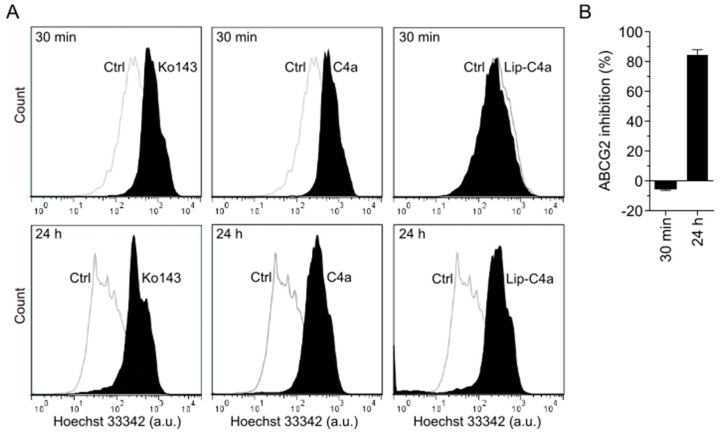
In vitro studies of ABCG2 inhibition using Lip-**C4a** (**C4a** loaded into liposomes composed of DSPC/DOPE). Cell-based transport assay using stably transfected cell lines overexpressing ABCG2. Hoechst 33342 (3 µM) and Ko143 (1 µM) were used as substrate and reference inhibitor, respectively. **C4a** or Lip-**C4a** were used at 10 µM. (**A**) Representative histograms were obtained by flow cytometry (30 min of incubation with inhibitor-upper part; 24 h of incubation with inhibitor-bottom part). (**B**) Data of mean ± SD of three independent experiments.

The low aqueous solubility of chromones presents a challenge for preclinical studies. In attempting to solve this problem, a liposome-based strategy was performed. **C4a** was loaded into liposomes composed of distearoylphosphatidylcholine (DSPC) and dioleoylphosphatidylethanolamine (DOPE). This preparation was named as Lip-**C4a** and tested as a drug delivery system of chromones. The liposomal drug incorporation and delivery were evaluated using the cell-based transport approach by flow cytometry using stably transfected cells overexpressing ABCG2 (HEK293-*ABCG2*). As shown in Figure 6A, Lip-**C4a** did not inhibit the ABCG2-mediated transport of the substrate hoechst 33342. In contrast, **C4a** in DMSO was used as a control, producing a similar inhibition effect to the reference inhibitor Ko143 (Figure 6A). To evaluate a possible time-dependent effect, Lip-**C4a** was tested after 24 h of cell exposure. Interestingly, Lip-**C4a** completely inhibited the ABCG2 efflux after this period, suggesting a time-dependent effect of either cell incorporation of the liposomes or **C4a** release from the liposomes (Figure 6A,B).

### 3.5. G. intestinalis and Human Extracellular Vesicles (EVs) for Delivery of Chromone (***C4a***)

Since liposomes efficiently deliver **C4a** to the target, an innovative lipid-based strategy based on EVs was tested. Despite several advantages of using liposomes as drug delivery vehicles, the use of EVs was proposed to circumvent the Achilles heel of synthetic lipids, that might include adverse side effects, mostly mediated by the immune response [41].

**Figure 7:**
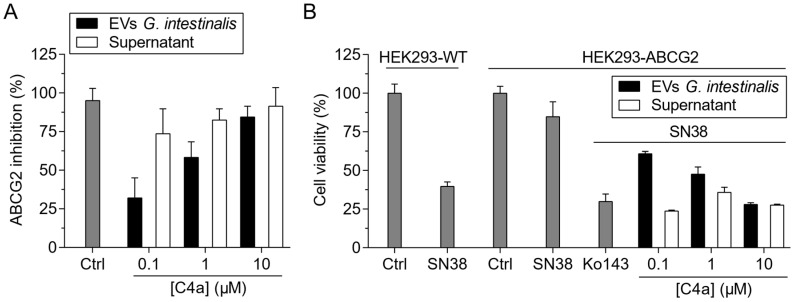
In vitro studies of ABCG2 inhibition using EVs *G. intestinalis*-**C4a** (**C4a** loaded into EVs from *G. intestinalis*). Cell-based transport assay using stably transfected cell lines overexpressing ABCG2. Hoechst 33342 (3 µM) and Ko143 (1 µM) were used as substrate and reference inhibitor, respectively. (**A**) **C4a** free in the supernatant or EVs *G. intestinalis*-**C4a** were tested at 0.1, 1 and 10 µM. Data of mean ± SD of three independent experiments. (**B**) Cell viability assay on wild-type cells (HEK293-WT) and HEK293-*ABCG2* cells. SN38 (10 nM) was used as chemotherapeutic drug transported by ABCG2. Ko143 (1 µM) was used as a reference inhibitor. Co-treatment of SN38 with either **C4a** free in the supernatant or EVs *G. intestinalis*-**C4a** were tested at 0.1, 1 and 10 µM. Data of mean ± SD of three independent experiments.

Firstly, EVs from trophozoites of *Giardia intestinalis* were obtained after incubation at 37 °C with CaCl_2_. *G. intestinalis* was chosen for the well-known high yield of EVs released after stimulation. To evaluate the drug incorporation into EVs of *G. intestinalis*, these EVs were incubated for 30 min with the fluorescent dye hoechst 33342. The non-incorporated remaining dye was removed by ultracentrifugation. EVs before and after ultracentrifugation were evaluated by flow cytometry, demonstrating that hoechst 33342 was successfully incorporated into the EVs or adsorbed on the lipid surface (Figure S5). This additional step of centrifugation was performed to completely remove the solvent used for drug solubilization, in the case of **C4a**, DSMO. As proof of concept of the use of EVs as the carrier of ABCG2 inhibitors, a range of **C4a** from 0.1 to 10 µM was incubated with EVs from *G. intestinalis*. As shown in Figure 7A, EVs containing the inhibitor (EVs *G. intestinalis*-**C4a**) caused a dose-dependent inhibition. These data revealed that **C4a** was incorporated into EVs from *G. intestinalis* after incubation and these EVs efficiently delivered **C4a** to HEK293-*ABCG2* cells. In addition, the control performed with the supernatant containing **C4a** free also inhibited ABCG2 transport activity, suggesting that the drug incorporation was partial (Figure 7A).

To confirm the functional inhibition of ABCG2, an in vitro chemosensitization assay was performed. The cytotoxic effect of the active metabolite of the chemotherapeutic irinotecan (SN38), that is transported by ABCG2, was evaluated in wild-type HEK293 and HEK293-*ABCG2* cells. As shown in Figure 7B, SN38 was highly cytotoxic on wild-type cells. A mild decrease in the cell viability was observed on cells overexpressing ABCG2, confirming that SN38 is transported by ABCG2. The co-treatment by the combination of SN38 and Ko143 completely sensitized the cells overexpressing ABCG2 (Figure 7B). In addition, the co-treatment of SN38 with EVs of *G. intestinalis* containing increasing concentrations of **C4a** (0.1 to 10 µM) also sensitized ABCG2 cells. A complete reversion of the resistance phenotype mediated by ABCG2 was observed using EVs of *G. intestinalis* incorporated with 10 µM of **C4a** or the supernatant containing **C4a** free at 0.1 to 10 µM (Figure 7B). Together, these data represent a proof of concept that biological vesicles are powerful tools to incorporate and promote the delivery of hydrophobic drugs.

To overcome the evident biocompatibility disadvantage of clinical applications of EVs of *G. intestinalis* as vehicles for drug delivery, we propose the use of EVs from human blood. Human EVs were obtained by two different approaches following blood collection from healthy donors: (1) circulating EVs from blood plasma and (2) EVs from blood cells after stimulation using CaCl_2_, as performed for *G. intestinalis.* The empty human EVs were analyzed by nanoparticle tracking analysis (NTA) and dynamic light scattering (DLS). EVs from human plasma showed a smaller size than those obtained from blood cells (Figure S6A–C). However, the concentration was lower for EVs from blood cells after stimulation with CaCl_2_ (Figure S6D). NTA and DLS analysis confirmed the integrity of the EVs after isolation. In addition, both EVs showed sizes that might correspond to large vesicles (average ≥ 125 nm) and there was no significant change in the size of EVs between the empty and drug-loaded EVs (Figure S6E). In addition, 0.4 µg/µL of albumin was detected in samples of circulating EVs from plasma by an immunoturbidimetric method. Considering the total protein amount of 56 µg/µL determined by Bradford assay, the presence of albumin corresponds to approximately 2%. Thus, specific applications in the absence of protein contaminants, such as albumin and ApoB-100, might require additional purification steps.

**Figure 8:**
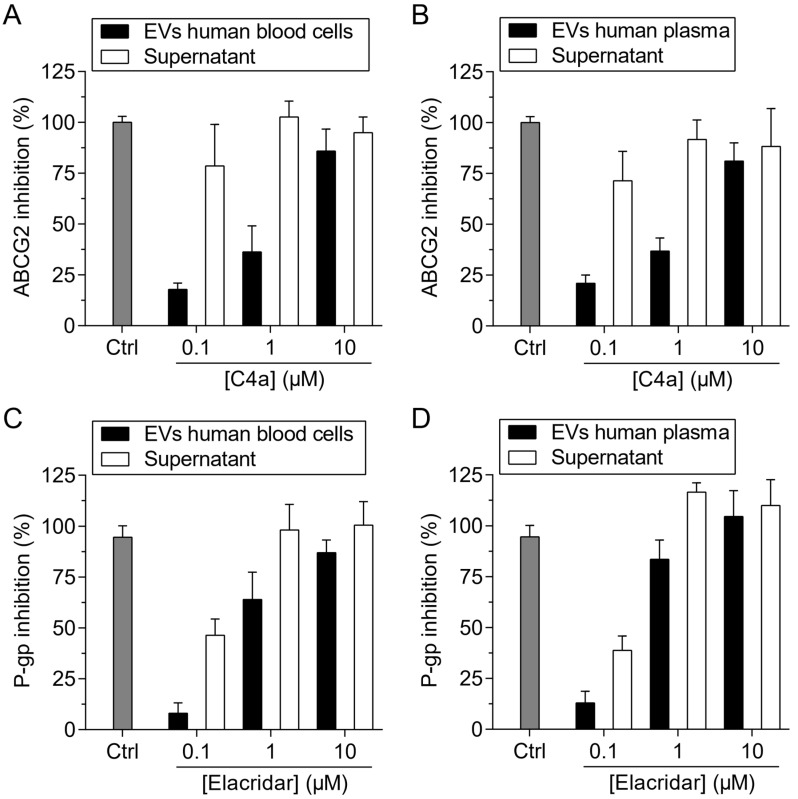
In vitro studies of ABCG2 and P-gp inhibition using EVs *human*-**C4a** (**C4a** loaded into EVs from blood human cells and plasma). Cell-based transport assay using stably transfected cell lines overexpressing ABCG2 and P-gp. Data of mean ± SD of three independent experiments. For ABCG2, hoechst 33342 (5 µM) and **C4a** (1 µM) were used as substrate and reference inhibitor, respectively. (**A**) **C4a** free in the supernatant or EVs human blood cells-**C4a** were tested at 0.1, 1 and 10 µM. (**B**) **C4a** free in the supernatant or EVs human plasma-**C4a** were tested at 0.1, 1 and 10 µM. For P-gp, rhodamine 123 (10 µM) and elacridar (1 µM) were used as substrate and reference inhibitor, respectively. (**C**) Elacridar free in the supernatant or EVs human blood cells–elacridar were tested at 0.1, 1 and 10 µM. (**D**) Elacridar free in the supernatant or EVs human plasma–elacridar were tested at 0.1, 1 and 10 µM.

EVs from human blood cells were incubated with a range of **C4a** from 0.1 to 10 µM. EVs containing the inhibitor (EVs human blood-**C4a**) inhibited the hoechst 33342 transport mediated by ABCG2 in a concentration-dependent manner (Figure 8A). As previously observed, the supernatant containing **C4a** free strongly inhibited ABCG2, even in the lower concentration tested (0.1 µM). To evaluate the use of blood-circulating EVs, the EVs from plasma were also loaded with **C4a**. Interestingly, the same results were observed (Figure 8B). Together, these data demonstrate that human EVs can be used for drug delivery of inhibitors and highlight the use of plasma-circulating EVs, with the advantage of easy and rapid obtention. In order to broaden the feature of EVs, we used the same approach for the incorporation and delivery of the P-gp inhibitor, elacridar. As shown in Figure 8C,D, human blood and plasma EVs incorporated and delivered elacridar to cells inhibiting the transport of rhodamine 123 mediated by P-gp.

## 4. Discussion

The development of potent inhibitors of ABCG2 as a strategy to tackle the multidrug resistance to chemotherapy represents a valuable approach. In this regard, hundreds of ABCG2 inhibitors were reported, belonging to diverse chemical classes and without sharing specific chemical features. However, it is admitted that hydrophobicity is a common characteristic [8]. Chromone derivatives were reported as inhibitors of P-gp and ABCG2 a few years after the discovery of ABCG2 [42,43]. These inhibitors were optimized and perfected to provide MBL-II-141 as the first chromone to enter the preclinical trials [19,22]. Further pharmacomodulation provided the chromone **C4a** as one of the strongest inhibitors of ABCG2 so far reported [21]. Many of the features of **C4a** highlight the need to carry out further preclinical and clinical studies on this inhibitor: its ease of synthesis allowing the scale-up process, its safety as evidenced by its innocuity, the high inhibition effect and its selectivity toward ABCG2 versus P-gp and MRP1. Moreover, evidence about its binding site and binding mode has been obtained, thanks to bioinformatics and molecular modelling. As observed for many other ABCG2 inhibitors [8], **C4a** also interacts with the amino acid residues V546, P439 and N436 (Figure 5).

In this work, we observed that in silico approaches should be carefully analyzed, since different ABC transporters can accommodate **C4a** in the binding pocket (Figure 2). Despite the stimulating effect caused by **C4a** on the ATPase activity of ABCG2 and P-gp, this membrane-based assay was able to discriminate the affinity of this inhibitor toward ABCG2 (Figure 3). In general, it is expected that functional inhibitors of ABC transporters inhibit the ATPase activity. However, this stimulation of the ATPase activity caused by **C4a** was observed for many compounds from different classes, such as stilbenes [14], curcumin analogues [44], sildenafil [45] and others [8]. As expected, the transport assay confirmed the selectivity of **C4a** toward ABCG2 (Figure 4A). This selective effect was already described for other chromone derivatives, including MBL-II-141 [19,46]. Together, these data highlight the importance of cell-based transport assays for studies of ABC transporter inhibitors.

ABCG2 inhibitors are described to not be specific for a single substrate, but to inhibit the transport activity of protein, abrogating the efflux of different substrates without chemical structural correlation. Here, this feature was investigated, and the data confirmed that **C4a** is capable of inhibiting different substrates (Figure 4C,D), as recently described for indeno [1,2-*b*]indoles [17] and porphyrins [18]. **C4a** also triggered a 5D3-shift, an effect well documented for most ABCG2 inhibitors [47]. Despite the potential of **C4a**, this inhibitor suffers from its low solubility, as was the case for MBL-II-141 and many other ABCG2 inhibitors. Indeed, for the cellular tests, DMSO is a valuable solvent when used in small amounts. However, for preclinical studies this method is not desirable. In this study, we demonstrated the potential of lipid-based approaches for loading and delivery of **C4a**, starting from artificial lipid vesicles (liposomes) to human EVs.

Liposomes have been extensively used for the delivery of drugs. Many liposome-based drugs, including the chemotherapeutics doxorubicin, daunorubicin and vincristine, were approved by the FDA and are on the market [48]. As shown in Figure 6, liposomes containing **C4a** promoted a slow release of this ABCG2 inhibitor. The easy obtention and the inhibitory effect caused by Lip-**C4a** make this strategy highly promising for use in preclinical models, overcoming the problems observed during the in vivo studies using ABCG2 inhibitors [20,22].

In addition to liposomes, other LNPs can be used for drug delivery. LNPs of biological origin include EVs. One of the most important sources of EVs is blood [49]. In blood plasma, circulating EVs are mainly derived from blood cells; however, EVs from a variety of cells can also be present in the bloodstream [50]. EVs from murine red blood cells (RBC) were described for the delivery of the hydrophilic drugs doxorubicin and vancomycin [51], and human RBC was described for the delivery of RNA drugs [52] and the hydrophobic drug camptothecin [53]. A few examples have demonstrated the applicability of circulating EVs as vehicles for the delivery of dopamine using murine serum [54] and the peptide BAY55-9837 using commercial human serum [55]. As a proof of concept, we used EVs from *G. intestinalis* to incorporate and deliver **C4a** to cells overexpressing ABCG2 (Figure 7). The potential use of EVs for delivery of ABC transporter inhibitors was demonstrated using human EVs from stimulated blood cells and circulating plasma EVs. As shown in Figure 8, these EVs loaded with **C4a** or elacridar efficiently inhibited the ABCG2 or P-gp activity. These results open a new avenue for the use of human EVs in personalized medicine targeting ABC transporters.

## 5. Conclusions

In this study, we described the mechanism of inhibition caused by **C4a**, one of the strongest inhibitors of ABCG2 so far reported. This inhibitor acts selectively on ABCG2 by restoring the cell sensitivity against antitumor drugs that are ABCG2 substrates. The mechanistic study and molecular modelling allowed the rationalization of the inhibition mode of the title inhibitor. Despite the therapeutic potential of **C4a** to be used in combination with conventional anticancer drugs and to make the latter active again, the use of the inhibitor in preclinical and clinical trials was hampered by its low solubility. Any chemical modification attempting to solve the water solubility issues failed to provide reasonably solubility and maintain acceptable biological activity. Here, we report a new and highly efficient formulation method based on EVs to tackle the solubility issue. A robust experimental protocol using human plasma was developed to maintain a good balance between solubility and activity. Taken together, the data reported here are very encouraging for further preclinical and clinical development and this will be our main objective in the next phase of this work.

## 6. Patents

Close compounds were patented by us under the Brazilian patent N°: BR102012026574B1, published on 21 July 2021.

## Data Availability

The data that support the findings of this study are available from the corresponding author upon request.

## Supplementary Materials

The following supporting information can be downloaded at: https://www.mdpi.com/article/10.3390/pharmaceutics15041259/s1. Figure S1. Effect of Ko143 on ATPase activity using High-Five ABCG2 total membrane vesicles. Data represent the mean ± SD of three independent experiments performed in duplicate. Figure S2. (A–C) Frequency of interactions observed along with the molecular dynamic simulations. Each bar represents the mean and standard error of at least five independent simulations of 200 ns each, individual frequency values per simulation are depicted as spheres. Hydrogen bond interactions are represented by purple bars, hydrophobic by yellow, π-π interactions by orange and water bridges by light blue. (D–E) ligand RMSD along the analysed simulation calculated about the original initial conformation ligand’s heavy atoms, each simulation replicate is depicted in a different colour. Individual ligands are labelled as follow: SN38 (A and D); Ko143 (B and E) and C4a (C and F). Figure S3. Distances between the TMD and NBD are different between systems. Lateral and top view of the ABCG2 transporter (A and B, respectively), where the reference points for the TMD and the NBD in different chains, Phe439 (red) and Tyr463 (orange), respectively are represented as coloured spheres. Distance values found in the initial conformation are depicted as lines. (C) Violin plot representing the distribution of distances between the centre of mass for the Phe439 of each chain (red) and Tyr463 for all simulated ligands in comparison with the apo structure. Figure S4. Mean residue fluctuations obtained from the root mean square deviation fluctuations (RMSF) of the ABCG2’s backbone atoms calculated in relation to the initial simulation frame in comparison to simulations with the co-crystallized ligand, for each inhibitor as described in the labels, separated by Chain A (A) and Chain B (B). Each dark-coloured line represents the mean distance of the five independent (200 ns) simulations and the respective light-coloured dashed line represent the observed standard deviation. Black lines are the mean for the simulations with the apo structure. Blue shaded region (residues 350-375) represents the bundle helices in the NBD (C), which have shifts in fluctuation during simulations with our ligands in comparison to the apo structure, even after equilibration. Figure S5. EVs from trophozoites of Giardia lamblia incubated with hoechst 33342 (3 μM) for a period of 30 minutes. Two conditions were compared after hoechst 33342 loading: EVs before and after ultracentrifugation. The fluorescence of hoechst 33342 inside the EVs was monitored by flow cytometry. Figure S6. Analysis of EVs by nanoparticle tracking analysis (NTA) and dynamic light scattering (DLS). Representation of raw data of EVs derived from (A) blood cells and (B) plasma diluted in PBS (1:80) obtained by nanoparticle tracking analysis (NTA, Nanosight, Malvern, U.K.) (C) Mean size values of EVs derived from blood cells and plasma obtained by NTA. (D) Normalized values of EVs concentration derived from blood cells and plasm obtained by NTA. (E) Mean values of the size of the empty EVs and EVs after treatment with C4a and elacridar. EVs from blood cells and plasma were diluted in PBS (1:200) and the data obtained by dynamic light scattering (DLS, Zeta Sizer Nano Series, Malvern, UK).
