# Supplementary material for: Characterization of Potent ABCG2 Inhibitor Derived from Chromone: From the Mechanism of Inhibition to Human Extracellular Vesicles for Drug Delivery
Source: Pharmaceutics. 2023 Apr 17;15(4):1259. doi: 10.3390/pharmaceutics15041259 (PMC10144134; doi:10.3390/pharmaceutics15041259)
Supplement: Supplementary file 1 [file pharmaceutics-15-01259-s001.zip › pharmaceutics-2208962-supplementary.pdf]

Supplementary Materials

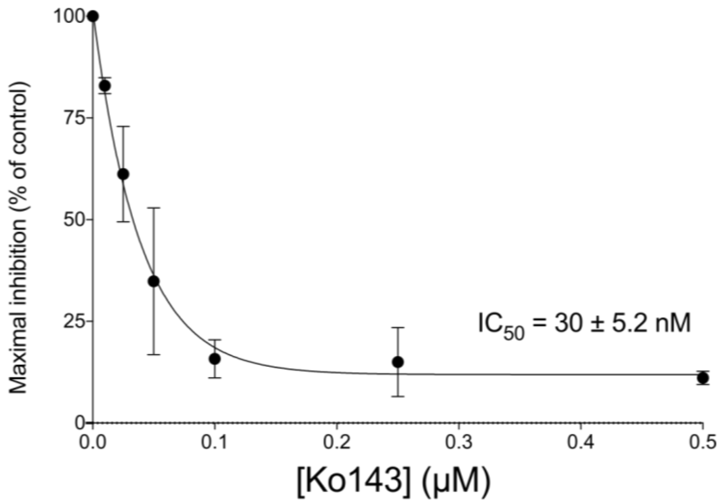

Figure S1. Effect of Ko143 on ATPase activity using High-Five ABCG2 total membrane vesicles. Data represent the mean ± SD of three independent experiments performed in duplicate.

Extended materials and methods

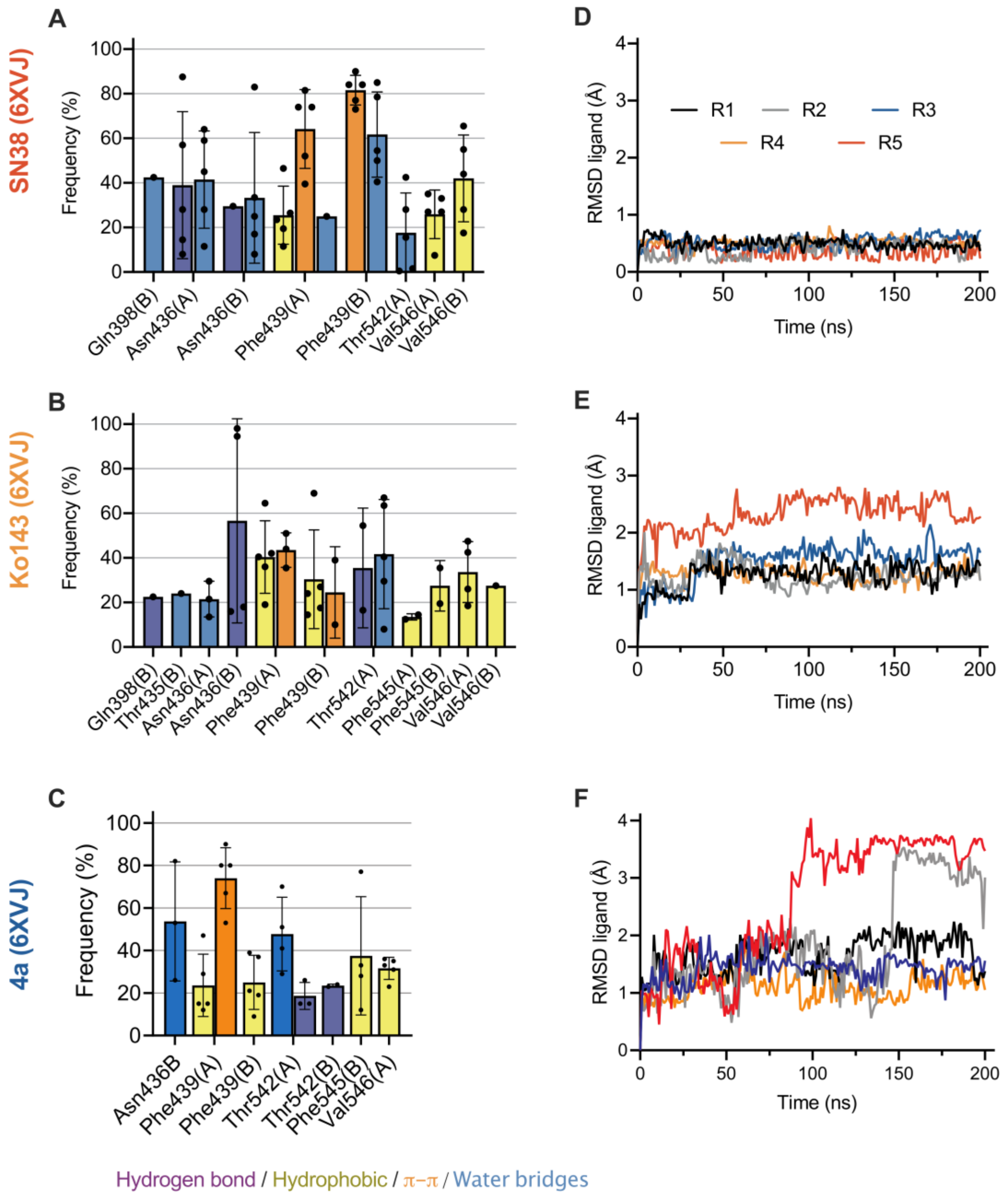

Figure S2. (A–C) Frequency of interactions observed along with the molecular dynamic simulations. Each bar represents the mean and standard error of at least five independent simulations of 200 ns each, individual frequency values per simulation are depicted as spheres. Hydrogen bond interactions are represented by purple bars, hydrophobic by yellow, π-π interactions by orange and water bridges by light blue. (D,E) ligand RMSD along the analysed simulation calculated about the original initial conformation ligand’s heavy atoms, each simulation replicate is depicted in a different colour. Individual ligands are labelled as follow: SN38 (A,D); Ko143 (B,E) and **C4a** (C,F).

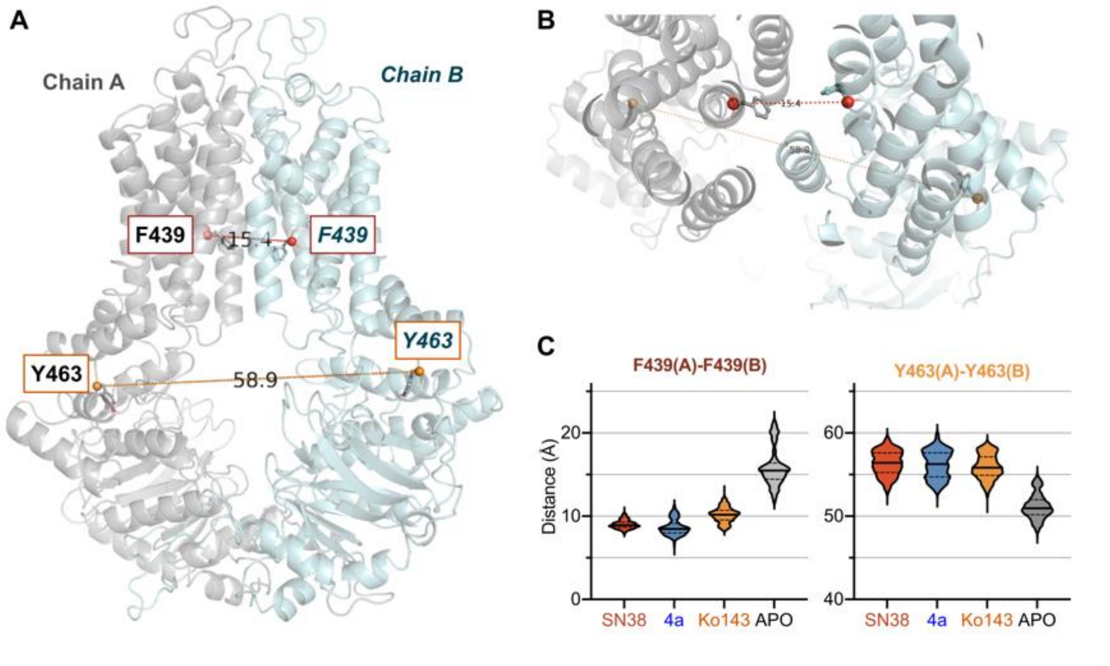

Figure S3. Distances between the TMD and NBD are different between systems. Lateral and top view of the ABCG2 transporter (A and B, respectively), where the reference points for the TMD and the NBD in different chains, Phe439 (red) and Tyr463 (orange), respectively are represented as coloured spheres. Distance values found in the initial conformation are depicted as lines. (C) Violin plot representing the distribution of distances between the centre of mass for the Phe439 of each chain (red) and Tyr463 for all simulated ligands in comparison with the apo structure.

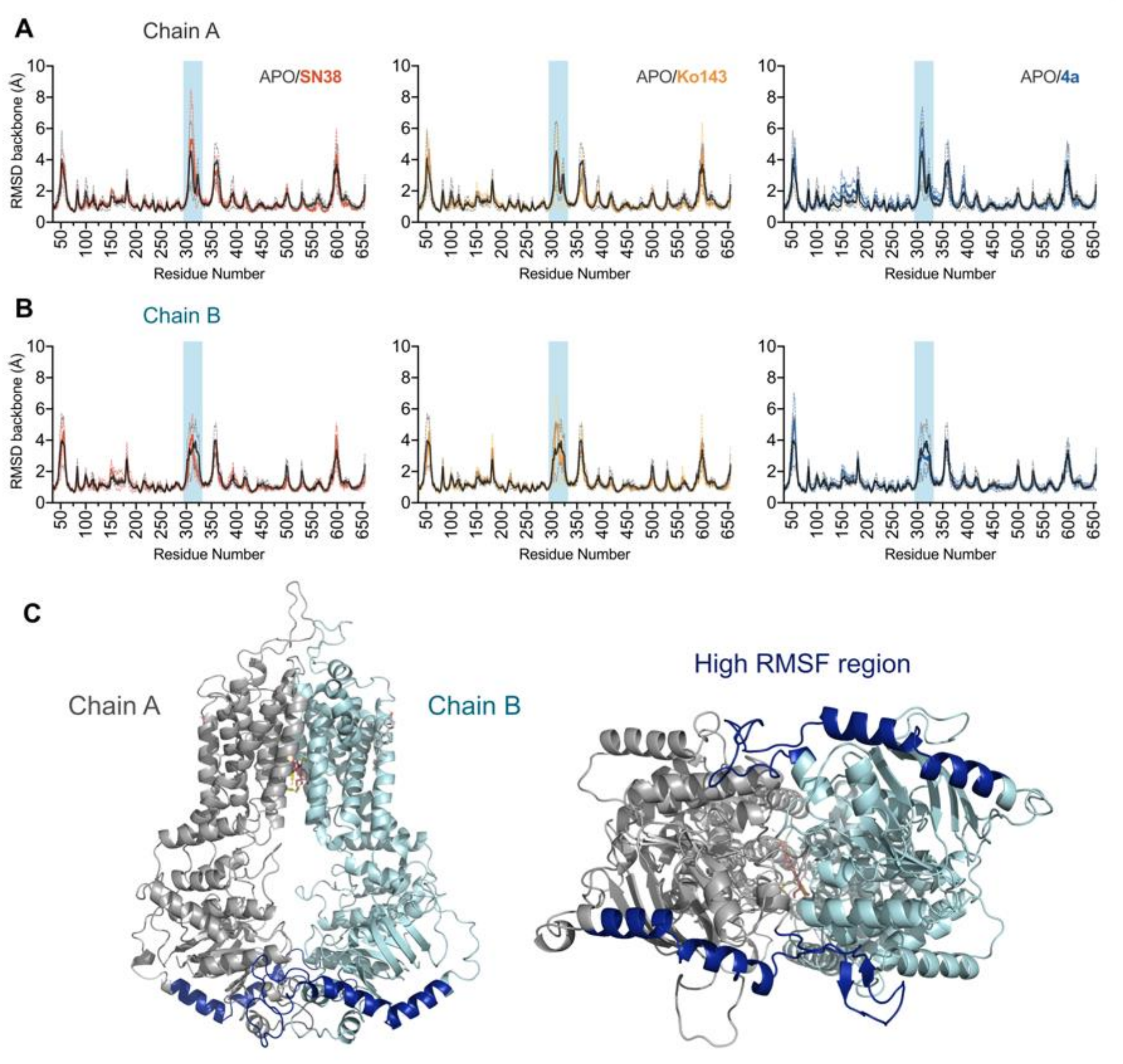

Figure S4. Mean residue fluctuations obtained from the root mean square deviation fluctuations (RMSF) of the ABCG2's backbone atoms calculated in relation to the initial simulation frame in comparison to simulations with the co-crystallized ligand, for each inhibitor as described in the labels, separated by Chain A (A) and Chain B (B). Each dark-coloured line represents the mean distance of the five independent (200 ns) simulations and the respective light-coloured dashed line represent the observed standard deviation. Black lines are the mean for the simulations with the apo structure. Blue shaded region (residues 350-375) represents the bundle helices in the NBD (C), which have shifts in fluctuation during simulations with our ligands in comparison to the apo structure, even after equilibration.

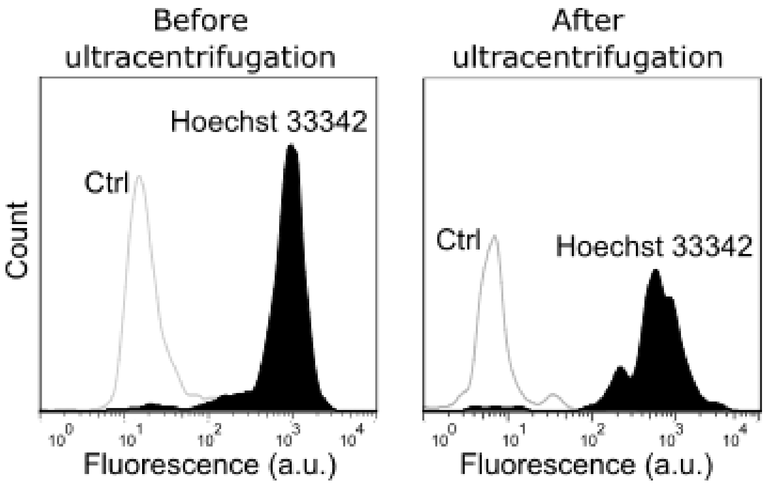

Figure S5. EVs from trophozoites of *Giardia lamblia* incubated with hoechst 33342 (3 μM) for a period of 30 minutes. Two conditions were compared after hoechst 33342 loading: EVs before and after ultracentrifugation. The fluorescence of hoechst 33342 inside the EVs was monitored by flow cytometry.

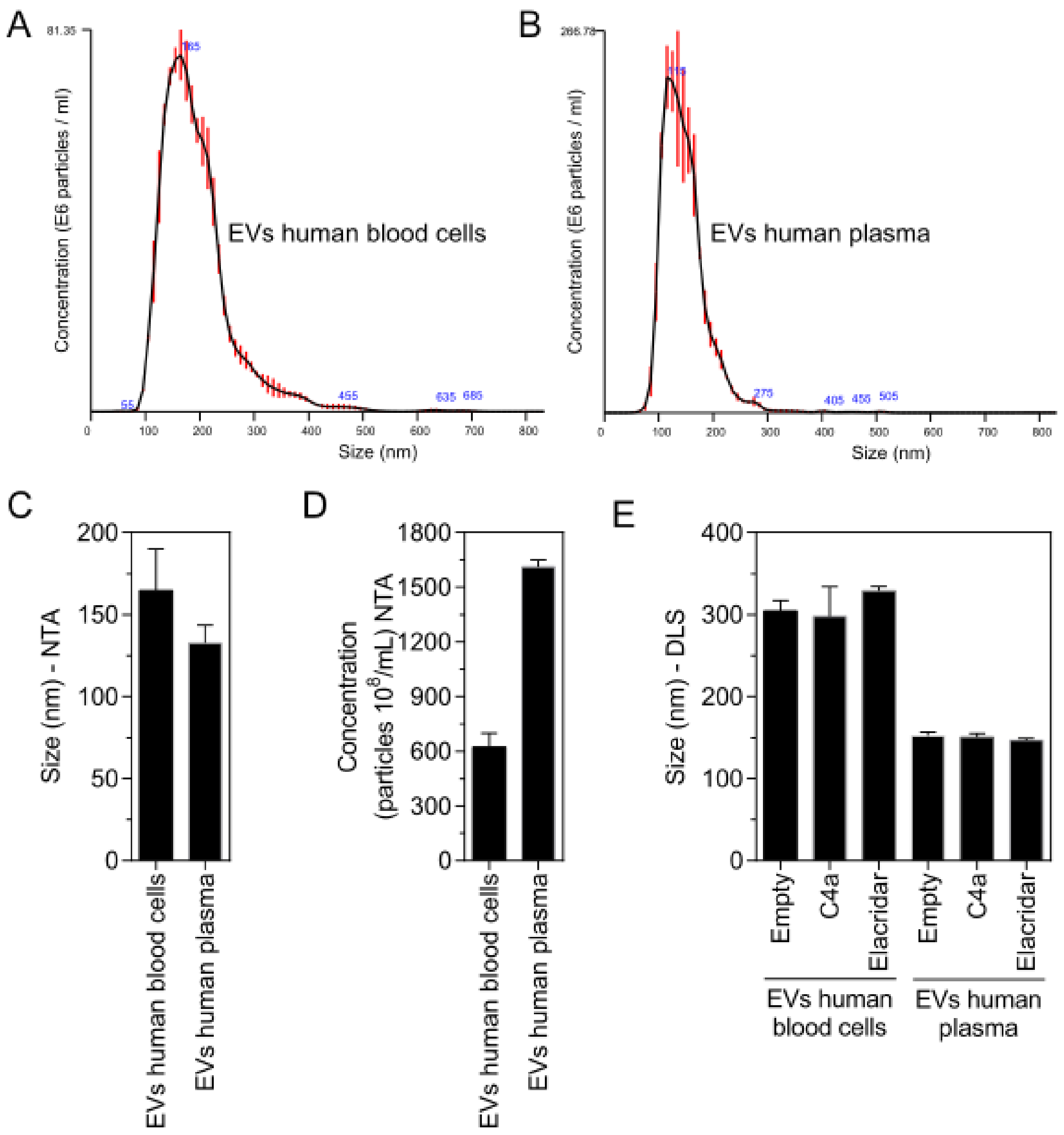

Figure S6. Analysis of EVs by nanoparticle tracking analysis (NTA) and dynamic light scattering (DLS). Representation of raw data of EVs derived from (A) blood cells and (B) plasma diluted in PBS (1:80) obtained by nanoparticle tracking analysis (NTA, Nanosight, Malvern, U.K.) (C) Mean size values of EVs derived from blood cells and plasma obtained by NTA. (D) Normalized values of EVs concentration derived from blood cells and plasm obtained by NTA. (E) Mean values of the size of the empty EVs and EVs after treatment with **C4a** and elacridar. EVs from blood cells and plasma were diluted in PBS (1:200) and the data obtained by dynamic light scattering (DLS, Zeta Sizer Nano Series, Malvern, UK).
